# Ultra-High-Resolution Time-of-Flight MR-Angiography for the Noninvasive Assessment of Intracranial Aneurysms, Alternative to Preinterventional DSA?

**DOI:** 10.1007/s00062-023-01320-z

**Published:** 2023-07-04

**Authors:** Tilman Schubert, Hakim Shakir Husain, Patrick Thurner, Jawid Madjidyar, Isabelle Barnaure, Marco Piccirelli, Markus Klarhöfer, Michaela Schmidt, Peter Speier, Christoph Forman, Zsolt Kulcsar

**Affiliations:** 1 Department of Neuroradiology, University Hospital Zurich, Zurich, Switzerland; 2 Siemens Healthineers, Zurich, Switzerland; 3 Siemens Healthineers, Erlangen, Germany; 4 Baby Memorial Hospital, Calicut, Kerala India; 5 Parco Institute of Medical Sciences, Vatakara, Kerala India; 6 Neo Hospital, Noida, Uttar Pradesh India

**Keywords:** MR-angiography, Non-invasive assessment, Compressed sensing, Digital subtraction angiography, Pre-treatment, Post-treatment

## Abstract

**Purpose:**

The 3D time-of-flight (TOF) magnetic resonance angiography (MRA) at 3T shows high sensitivity for intracranial aneurysms but is inferior to three-dimensional digital subtraction angiography (3D-DSA) regarding aneurysm characteristics. We applied an ultra-high-resolution (UHR) TOF-MRA using compressed sensing reconstruction to investigate the diagnostic performance in preinterventional evaluation of intracranial aneurysms compared to conventional TOF-MRA and 3D-DSA.

**Methods:**

In this study 17 patients with unruptured intracranial aneurysms were included. Aneurysm dimensions, configuration, image quality and sizing of endovascular devices were compared between conventional TOF-MRA at 3T and UHR-TOF with 3D-DSA as gold standard. Quantitatively, contrast-to-noise ratios (CNR) were compared between TOF-MRAs.

**Results:**

On 3D-DSA, 25 aneurysms in 17 patients were detected. On conventional TOF, 23 aneurysms were detected (sensitivity: 92.6%). On UHR-TOF, 25 aneurysms were detected (sensitivity: 100%). Image quality was not significantly different between TOF and UHR-TOF (*p* = 0.17). Aneurysm dimension measurements were significantly different between conventional TOF (3.89 mm) and 3D-DSA (4.2 mm, *p* = 0.08) but not between UHR-TOF (4.12 mm) and 3D-DSA (*p* = 0.19). Irregularities and small vessels at the aneurysm neck were more frequently correctly depicted on UHR-TOF compared to conventional TOF. Comparison of the planned framing coil diameter and flow-diverter (FD) diameter revealed neither a statistically significant difference between TOF and 3D-DSA (coil *p* = 0.19, FD *p* = 0.45) nor between UHR-TOF and 3D-DSA (coil: *p* = 0.53, FD 0.33). The CNR was significantly higher in conventional TOF (*p* = 0.009).

**Conclusion:**

In this pilot study, ultra-high-resolution TOF-MRA visualized all aneurysms and accurately depicted aneurysm irregularities and vessels at the base of the aneurysm comparably to DSA, outperforming conventional TOF. UHR-TOF with compressed sensing reconstruction seems to represent a non-invasive alternative to pre-interventional DSA for intracranial aneurysms.

## Introduction

Non-contrast time-of-flight magnetic resonance angiography (TOF-MRA) has become the standard of care in evaluation of unruptured intracranial aneurysms due to its high specificity and sensitivity as well as the lack of ionizing radiation. The limited spatial resolution and flow-related artifacts may, however, prevent detailed analysis of aneurysm morphology and of neighboring arteries [1]. Therefore, 3D digital subtraction angiography (DSA) is still considered the gold standard of treatment planning of intracranial aneurysms despite its invasive nature potentially associated with complications [2, 3]. A noninvasive, high spatial resolution imaging modality for this purpose would therefore be welcome in the diagnostic arsenal for cerebral aneurysms.

Compressed sensing (CS) is a method to accelerate MR image acquisition by k‑space undersampling and recovering the missing data through exploiting sparsity in an appropriate domain [4, 5]. CS has been applied to MRI preferentially to reduce scan time [6–9]. It can also be applied to increase spatial resolution while keeping scan time constant. The potential benefit of very high spatial resolution CS TOF MRA over conventional TOF MRA for treatment planning of intracranial aneurysms has however not yet been exploited.

The purpose of the present study was to compare the diagnostic performance of ultra-high-resolution (UHR) TOF MRA using compressed sensing reconstruction with conventional TOF MRA and the gold standard 3D-DSA in the preinterventional assessment of intracranial aneurysms to evaluate if UHR TOF may be an alternative to preinterventional DSA.

## Material and Methods

### Patients

A total of 17 patients (12 female, mean age 52 years) were included in this study. All patients signed a general informed consent form for retrospective research at the University Hospital Zurich. The study was approved by the regional ethics committee. All patients were diagnosed with an incidental intracranial aneurysm and had received diagnostic work-up including a 3T MRI examination. All patients received a dedicated MRI examination including an ultra-high-resolution CS TOF the day prior to treatment. The interval from initial MRI to UHR-TOF ranged from 3 to 207 days (average: 90 days). The UHR-TOF was performed 1 day prior to DSA. The interval from conventional TOF to DSA ranged from 4 to 208 days (average: 90.1 days).

### MR Imaging Parameters

All UHR-TOF examinations were performed on a MAGNETOM Skyra 3T MR imaging scanner (Siemens Healthineers, Erlangen, Germany) using a 20-channel head-neck coil and a prototype sequence (ASP 1005E Sparse MRA [10] Siemens Healthineers). Scanning parameters were TR 21.6 ms, TE 3.3 ms, flip angle (FA) 18°, acquisition matrix 544/494, field of view 218/198 mm, slice thickness 0.27 mm, resulting in an acquired spatial resolution of 0.4 × 0.4 × 0.27 mm^3^ that was reconstructed to an isotropic 0.3 × 0.3 × 0.3 mm^3^ voxel size. The compressed sensing acceleration factor of UHR-TOF was set at 10.4. The total acquisition time was 5 min and 43 s.

Of the conventional TOF acquisitions, 10 were performed at our institution either on a MAGNETOM Skyra 3T (Siemens Healthineers) in 7 cases or on a Philips Ingenia 3T (Philips Medical, Best, Netherlands) in 3 cases. The remaining seven patients were scanned at referring institutions on a Philips Ingenia 3T (three cases), Philips Achieva 3T (two cases), Siemens Healthineers Skyra 3T and a GE Signa 3T (GE medical, Waukeesha, WI, USA) in one case each.

Mean in-plane resolution of the conventional TOF acquisition was 0.53 × 0.67 mm (range 0.35–0.6 × 0.57–0.8 mm) with an average slice thickness of 0.6 mm (range 0.55–0.7 mm).

All imaging parameters for conventional TOF examinations are listed in Table 1.

**Table 1:** Summary of the FOV, matrix, slice thickness, TR, TE and flip angle (FA) of all conventional TOF acquisitions as well as the in-plane unreconstructed resolution. Patients scanned at the study institution are marked “Inhouse” and were scanned either on a Siemens Magnetom Skyra 3T or Philips Ingenia 3T

	Conventional TOF	FOVx	FOVy	Matrix x	Matrix y	In-plane resolution x	In-plane resolution y	Slice thickness	TR/TE (ms)	FA
Patient 1	Philips Achieva 3T	200	200	368	244	0.54	0.82	0.6	23/3.4	18
Patient 2	Siemens Skyra 3T	200	180	384	313	0.52	0.58	0.6	21/3.4	18
Patient 3	Siemens Skyra Inhouse	190	165	320	238	0.59	0.69	0.6	21/3.4	20
Patient 4	Philips Ingenia 3T	200	200	400	308	0.5	0.65	0.55	25/3.4	20
Patient 5	Philips Ingenia 3T	200	200	572	329	0.35	0.61	0.55	25/3.4	20
Patient 6	Philips Ingenia Inhouse	200	200	400	308	0.5	0.65	0.55	25/3.4	20
Patient 7	Philips Ingenia Inhouse	200	200	400	308	0.5	0.65	0.55	25/3.4	20
Patient 8	Philips Ingenia 3T	200	200	500	333	0.4	0.6	0.6	25/3.4	20
Patient 9	Philips Ingenia 3T	200	200	400	308	0.5	0.65	0.55	23/3.5	18
Patient 10	Siemens Skyra Inhouse	190	165	320	238	0.59	0.69	0.6	21/3.4	20
Patient 11	Philips Achieva 3T	200	200	368	244	0.54	0.82	0.6	25/6.2	20
Patient 12	Siemens Skyra Inhouse	190	165	320	238	0.59	0.69	0.6	21/3.4	20
Patient 13	GE Signa 3T	220	150	416	256	0.53	0.59	0.7	30/2.7	15
Patient 14	Siemens Skyra Inhouse	190	165	320	238	0.59	0.69	0.6	21/3.4	20
Patient 15	Siemens Skyra Inhouse	190	165	320	238	0.59	0.69	0.6	21/3.4	20
Patient 16	Siemens Skyra Inhouse	190	165	320	238	0.59	0.69	0.6	21/3.4	20
Patient 17	Siemens Skyra Inhouse	190	165	320	238	0.59	0.69	0.6	21/3.4	20

*FOV* Field-of-View, *TR* Relaxation Time, *TE* Echo Time, *ms* Milliseconds, *x* x-axis direction, *y* y-axis direction

### Image Evaluation

**Fig. 1:**
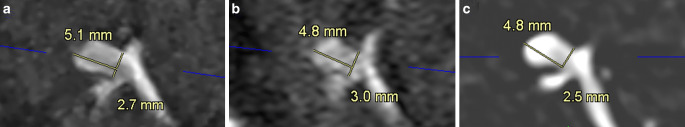
Representative measurements of the aneurysm height and largest diameter aneurysm neck on UHR-TOF (**a**), conventional TOF (**b**) and 3D-DSA (**c**). The aneurysm neck to the anterior communicating artery is more clearly demarcated on UHR-TOF and 3D-DSA compared to conventional TOF

Aneurysm dimensions were quantified on multiplanar reconstructions by a senior interventional neuroradiologist. Height, maximum and minimum diameters and maximum neck diameter in both MRA acquisitions and 3‑D DSA were measured side by side in all three modalities to adapt measurement orientations (Fig. 1). Measurements were performed on a commercially available PACS system (DeepUnity, Dedalus Healthcare, Bonn, Germany). For qualitative assessment and device sizing, images were assessed by two senior interventional neuroradiologists in consensus reading where also the number of aneurysms per patient was registered. To minimize bias, conventional TOF images were read first, followed by UHR-TOF and 3D-DSA with a minimum interval of 4 weeks between modalities. Image quality was assessed on a 3-point scale with 3 indicating completely blurred arteries and severe artifacts (poor, nondiagnostic), 2 indicating slight to moderate artifacts (sufficient) and 1 indicating excellent arterial delineation and no artifacts (excellent image quality). Aneurysm configuration (regular vs. irregular, daughter aneurysms, blebs) shape and small vessels originating at the aneurysm neck were registered as anatomical features. Regarding treatment type (coiling/flow diversion), the sizing of the framing coil or flow-diverter diameter were defined on each MRA acquisition and on DSA. In case both coiling and flow-diverter treatment were deemed feasible, both the framing coil diameter and flow-diverter diameter were recorded.

Contrast-to-noise ratios (CNR) were assessed by drawing a 3 mm^2^ ROI in the vertical petrous segment of the internal carotid artery and a 10 mm^2^ ROI in the temporal muscle on the same slice. The CNR was calculated using the following formula:

$$\text{CNR}=\frac{\text{SI}_\text{ica}-\text{SI}_\text{tm}}{\sqrt{{\text{StD}_\text{ica}}^{2}-{\text{StD}_\text{tm}}^{2}}}$$

Where SI_ica_ refers to the signal intensity in the internal carotid artery, SI_tm_ to the signal intensity in the temporal muscle and StD_ica_ refers to the standard deviation within the ROI in the internal carotid artery.

### Statistical Analysis

Aneurysm dimensions between the three modalities were analyzed using one-way ANOVA. The diameter of the framing coil or flow-diverter diameter were compared using either paired 2‑sided Studentʼs t‑test or Wilcoxon-rank test depending on normal distribution. Furthermore, the sensitivity regarding aneurysm detection compared to DSA was evaluated. In order to minimize bias derived from different MR systems, we compared aneurysm dimension measurements in the subgroup that received conventional TOF and UHR TOF at different MRI scanners and in patients that received both conventional TOF and UHR TOF at the same scanner.

Significance levels were set at 0.1. Statistical analyses were performed with SPSS Version 27 (IBM, Armonk, NY, USA).

## Results

**Fig. 2:**
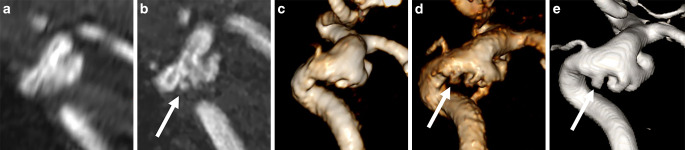
On ultra-high-resolution-time-of-flight (TOF) MRA (multiplanar [MPR], **b**) and volume rendering (VR, **d**) reconstructions), one small aneurysm adjacent to a larger inferior wall carotid artery aneurysm can be clearly separated (white arrows). On conventional TOF, the aneurysms appear as one broad-based aneurysm (MPR (**a**) and VR (**c**)). The additional aneurysm was confirmed on 3D-digital subtraction angiography (**e**, *arrow*). Conventional TOF parameters are TR/TE 23/3.4 ms, FA 18°, acquisition matrix 500/333, field of view 200 × 200 mm, slice thickness 0.6 mm

**Fig. 3:**
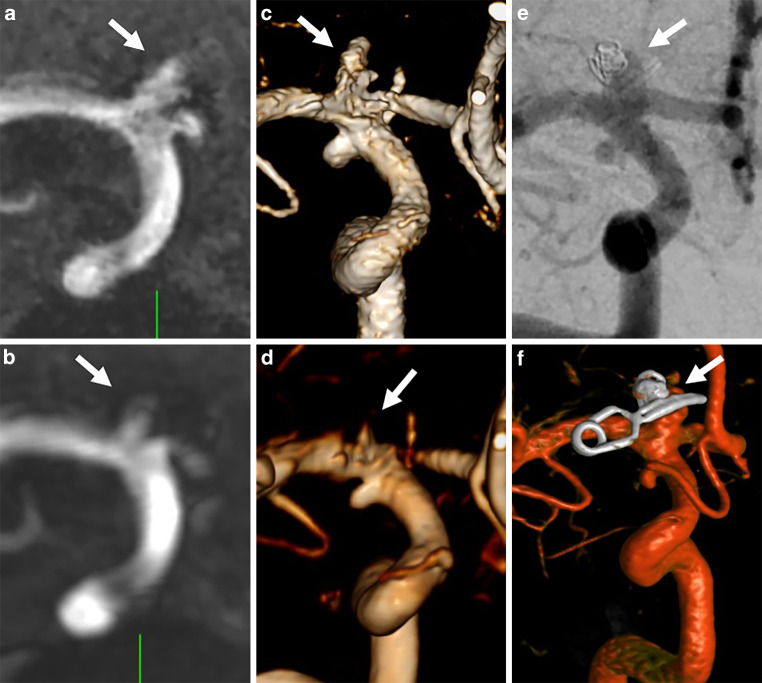
UHR-TOF shows a recurrent, previously clipped and coiled carotid‑T aneurysm in multiplanar (MPR) and volume rendered (VR) reconstructions (**a**, **c**, *arrows*), which was confirmed by DSA (**e**, **f**, *arrows*). The recurrence is not visible on the conventional TOF VR reconstruction and to a limited extent on the MPR reconstruction (**b**, **d**, *arrows*). Conventional TOF parameters are TR/TE 21/3.4 ms, FA 25°, acquisition matrix: 320/238, field of view: 190×165 mm, slice thickness 0.6 mm

On 3D digital subtraction angiography, 25 aneurysms were detected in 17 patients. On conventional TOF imaging, 23 aneurysms were detected (sensitivity: 92.6%), 2 aneurysms were not detected, 1 was a small aneurysm of the inferior wall of the internal carotid artery adjacent to a larger aneurysm (Fig. 2), the second was a recurrent, previously ruptured carotid T aneurysm treated by clipping and coiling (Fig. 3). Conventional TOF parameters of the missed aneurysm cases are added to the figure legends. On UHR-TOF imaging, 25 aneurysms were detected (sensitivity: 100%).

### Image Evaluation

#### Image Quality

There were no poor (nondiagnostic) examinations present in either group. Average image quality was rated 1.17 for conventional TOF and 1.06 for UHR-TOF (3-point scale with 3 indicating completely blurred arteries and severe artifacts (poor, nondiagnostic), 2 indicating slight to moderate artifacts (sufficient) and 1 indicating excellent arterial delineation and no artifacts (excellent image quality)).

#### Anatomical Features

**Fig. 4:**
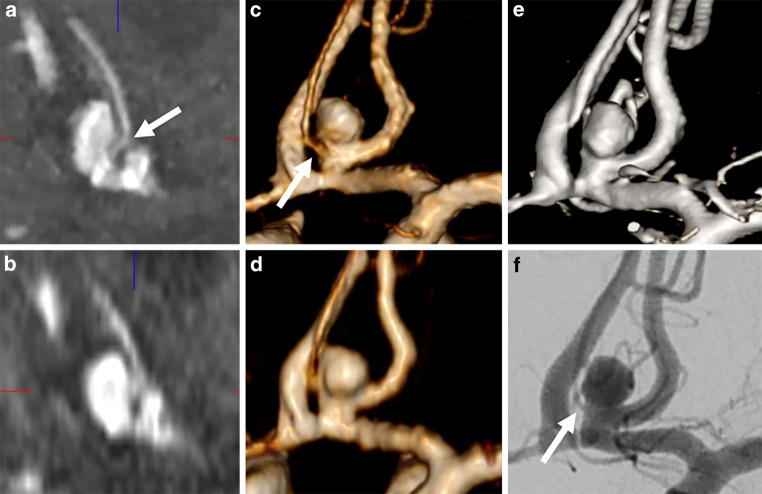
The origin of the common pericallosal artery variant at the aneurysm neck is visualized on UHR-TOF (**a**, **c**, *arrows*) but not on conventional TOF (**b**, **d**) and 3D-DSA (**e**). The vessel origin is confirmed on 2D DSA (**f**, *arrow*). Lack of visualization in 3D DSA is due to wash out from the contralateral A1 segment

Irregular shapes were registered in 10 out of 23 aneurysms on conventional TOF, in 13 out of 25 aneurysms on UHR-TOF and in 12 out of 25 aneurysms on 3D-DSA. Small vessels originating close to the aneurysm neck were detected on conventional TOF in 8 out of 23 aneurysms, on UHR-TOF in 13 out of 25 aneurysms and on 3D-DSA in 12 out of 25 aneurysms. One small vessel that was detected on UHR-TOF but not on 3D-DSA was confirmed on 2D DSA (Fig. 4).

#### Size Measurements

Mean aneurysm dimension measurements (height, maximum and minimum diameters, maximum neck diameters combined) were 4.2 mm on 3D-DSA 4.12 mm on UHR-TOF and 3.89 mm on conventional TOF. Average aneurysm height was 4.34 mm (range 2–11.8 mm) on 3D-DSA, 4.31 mm (range 1.2–11.5 mm) on UHR-TOF and 3.95 mm (range 2.5–11-2 mm) on TOF. Average aneurysm maximum diameters were 4.93 mm (range 1.7–11.4 mm) on 3D-DSA, 4.88 mm (range 1.5–13 mm) on UHR-TOF and 4.66 mm (range 1.7–12.9 mm) on TOF. Average minimum diameters were 4.1 mm (range 1.5–11.4 mm) on 3D-DSA, 3.9 mm (range 1.2–11.4 mm) on UHR-TOF and 3.74 mm (range 1.4–11.4 mm) on TOF. Average maximum neck diameters were 3.39 mm (range 1.3–9 mm) mm on 3D-DSA, 3.37 mm (range 1.3–9.2 mm) on UHR-TOF and 3.18 mm (range 1.3–9.3 mm) on TOF.

#### Framing Coil Size

A total of 18 aneurysms were judged to be primarily accessible for coiling, 5 for flow-diverter treatment and 2 aneurysms were evaluated to be amenable for both flow-diverter or coiling. Mean diameter of the framing coils decided upon was 3.925 mm (range 2–12 mm) based on 3D-DSA, 3.875 mm (range 2–12 mm) based on UHR-TOF and 4.05 mm (range 2–12 mm) based on conventional TOF.

#### Flow-diverter Diameter

Mean flow-diverter diameter chosen based on 3D-DSA was 4.39 mm (range 3.5–5 mm) mm, 4.25 mm (range 3–5 mm) mm based on UHR-TOF and 4.22 mm (range 3–5 mm) based on conventional TOF.

Quantitative CNR analysis:

Contrast-to-noise ratio calculations resulted in an average CNR of 1.03 (range 0.06–4.72, SD 1.01) for conventional TOF and an average CNR of 0.32 (range 0.05–0.92, SD 0.26) for UHR-TOF.

### Statistical Analysis

Shapiro-Wilk tests revealed a normal distribution of the aneurysm size measurements and a non-normal distribution of framing coil and flow-diverter dimensions. Therefore, a one-way analysis of variance was performed for comparison of the aneurysm dimensions and Wilcoxon-signed rank tests were performed to compare planned framing coil and flow-diverter sizes.

Comparison of aneurysm dimensions showed no statistically significant differences between 3D DSA, UHR-TOF and conventional TOF for largest neck diameter (*p* = 0.91), aneurysm height (*p* = 0.78), maximum diameter (*p* = 0.87) and minimum diameter (*p* = 0.81).

Comparison of the planned framing coil diameter revealed neither a statistically significant difference between TOF and 3D-DSA (*p* = 0.19) nor between UHR-TOF and 3D-DSA (*p* = 0.53).

Comparison of the planned flow-diverter diameter revealed neither a statistically significant difference between TOF and 3D-DSA (*p* = 0.45) nor between UHR-TOF and 3D-DSA (*p* = 0.33).

Statistical results are summarized in Table 2.

**Table 2:** Summary of mean values with standard deviations (in parentheses) of the aneurysm dimensions for conventional TOF, UHR-TOF and 3D-DSA. *P*-values resulting from the ANOVA (Analysis of Variance) for the comparison of parameters between conventional TOF, UHR-TOF and 3D-DSA are included

	TOF	UHR TOF	3D DSA	Difference between modalities
Aneurysm height (mm)	3.95 (2.3)	4.31 (2.0)	4.34 (2.14)	*P* = 0.91
Maximum diameter (mm)	4.66 (2.62)	4.88 (2.31)	4.93 (2.14)	*P* = 0.78
Minimum diameter (mm)	3.74 (2.4)	3.9 (2.17)	4.1 (2.13)	*P* = 0.87
Aneurysm neck (mm)	3.18 (2.02)	3.375 (1.76)	3.39 (1.69)	*P* = 0.81

Comparison of image quality revealed no significant difference between UHR-TOF and conventional TOF (*p* = 0.17).

Contrast-to-noise ratios of conventional TOF were significantly higher than CNR of UHR-TOF (*p* = 0.003).

#### Comparison between Scanners

No significant differences between aneurysm dimension measured with conventional TOF and URH TOF were found in either patients scanned with different MR scanners (*p* = 0.52–0.75) or with the same scanner (*p* = 0.39–0.77).

## Discussion

In this study, we evaluated the quantitative accuracy and preinterventional diagnostic performance of ultra-high-resolution TOF MR angiography with compressed sensing reconstruction compared to conventional TOF and 3D-DSA in a series of small aneurysms (average maximum diameter 5 mm). The main finding of the study was an equivalent sensitivity of UHR-TOF for intracranial aneurysms and accurate visualization of aneurysm features (irregularities, small vessels originating at the base) compared to 3D DSA. Conventional TOF failed to visualize two aneurysms (small aneurysm adjacent to another and recurrent aneurysm) and showed less anatomical aneurysm features than UHR-TOF.

The sizing of intrasaccular and extrasaccular devices was equally accurate with UHR-TOF and conventional TOF.

To our knowledge, no studies exist comparing the diagnostic performance of UHR-TOF and conventional TOF with DSA for preinterventional assessment of intracranial aneurysms.

In the present study, we were able to decrease the unreconstructed voxel size for the UHR-TOF roughly 5‑fold compared to conventional TOF at 3T. The optimized TOF sequence with compressed sensing reconstruction was described in a previous publication [11]. We showed that the increased spatial resolution of UHR-TOF MRA while keeping scan time in the range of conventional TOF enables visualization of aneurysm features that were not detected with conventional TOF at 3T.

The substantial increase in spatial resolution was achieved at the cost of a significantly decreased CNR. Interestingly, the higher spatial resolution and decreased CNR of UHR-TOF did not result in inferior image quality compared to conventional TOF.

The increased spatial resolution resulted in visualization of one small aneurysm, which, verified by 3D-DSA, changed the endovascular treatment approach from balloon-assisted coiling to placement of a flow-diverter (Fig. 2). In another case, a recurrent aneurysm next to coils and a microsurgical clip that was not detected on conventional TOF could clearly be visualized on UHR-TOF (Fig. 3). The improved visualization of aneurysms adjacent to foreign material introducing magnetic susceptibility is most likely due to less intravoxel dephasing in UHR-TOF because of the small voxel size.

On UHR-TOF, all cases of small arteries originating close to the aneurysm neck were correctly identified. This was superior to conventional TOF, where roughly two thirds of these vessels were visualized (Fig. 4). This feature aiding correct identification of small arteries originating at the aneurysm neck is important to assess the periprocedural risk of occluding these branches. Aneurysm irregularities were slightly underrecognized with conventional TOF and minimally overestimated with UHR-TOF.

No significant differences for aneurysm dimensions were found between modalities despite the smaller mean differences between UHR-TOF and DSA compared to conventional TOF and DSA. Accordingly, comparison between the size of intrasaccular coils and extrasaccular flow-diverters did not result in significant differences either. Although endovascular device sizing is performed based on invasive procedural imaging, potential sizes can be narrowed down based on both preinterventional ultra-high-resolution UHR-TOF but also conventional TOF.

Compressed sensing (CS) reconstruction has been successfully applied to TOF MR angiography in different neurovascular pathologies [4, 6–8, 10, 12–14]. Several publications have focused on the comparison of CS-TOF and conventional TOF MRA [6–8, 14, 15]. The general conclusions of these publications were a comparable image quality and clinical feasibility of CS-TOF compared to conventional TOF; however, no study is available that utilized compressed sensing reconstruction to heavily increase spatial resolution in order to evaluate its use in the evaluation of intracranial aneurysms.

The main limitation of the present study is that 7 out of 17 conventional TOF examinations were performed at external sites with different scanning parameters; however, all external conventional TOF examinations were performed on state of the art 3T-MRI scanners. Furthermore, the interval from conventional TOF to UHR-TOF was 90 days on average. Potentially, aneurysms might show small changes in size in this period.

In conclusion, ultra-high-resolution TOF MRA showed an equivalent performance in visualization of aneurysm characteristics compared to DSA in our study. Furthermore, all aneurysms were accurately diagnosed; therefore, UHR TOF has the potential to serve as a reliable and noninvasive alternative to preinterventional diagnostic angiography and outperformed conventional TOF in the present study; however, the results of this pilot study need to be confirmed in larger series.

## Abbreviations

CS: Compressed sensing
DSA: Digital subtraction angiography
FD: Flow-diverter
MRA: Magnetic resonance angiography
UHR: Ultra-high resolution
